# Expression and Activity of the BioH Esterase of Biotin Synthesis is Independent of Genome Context

**DOI:** 10.1038/s41598-017-01490-0

**Published:** 2017-05-19

**Authors:** Xinyun Cao, Lei Zhu, Zhe Hu, John E. Cronan

**Affiliations:** 1 Department of Microbiology, Urbana, Illinois 61801 USA; 2 Department of Biochemistry, University of Illinois, Urbana, Illinois 61801 USA

## Abstract

BioH is an α/β-hydrolase required for synthesis of the pimelate moiety of biotin in diverse bacteria. The *bioH* gene is found in different genomic contexts. In some cases (e.g., *Escherichia coli*) the gene is not located within a biotin synthetic operon and its transcription is not coregulated with the other biotin synthesis genes. In other genomes such as *Pseudomonas aeruginosa* the *bioH* gene is within a biotin synthesis operon and its transcription is coregulated with the other biotin operon genes. The esterases of pimelate moiety synthesis show remarkable genomic plasticity in that in some biotin operons *bioH* is replaced by other α/ß hydrolases of diverse sequence. The “wild card” nature of these enzymes led us to compare the paradigm “freestanding” *E. coli* BioH with the operon-encoded *P. aeruginosa* BioH. We hypothesized that the operon-encoded BioH might differ in its expression level and/or activity from the freestanding BioH gene. We report this is not the case. The two BioH proteins show remarkably similar hydrolase activities and substrate specificity. Moreover, *Pseudomonas aeruginosa* BioH is more highly expressed than *E. coli* BioH. Despite the enzymatic similarities of the two BioH proteins, bioinformatics analysis places the freestanding and operon-encoded BioH proteins into distinct clades.

## Introduction

Biotin, also known as vitamin H, is an essential enzyme cofactor found in all three domains of life. The cofactor is necessary for critical steps of central metabolism including fatty acid synthesis and amino acid degradation^1^. De novo synthesis of biotin is restricted to archea, bacteria, plants and a few fungi^2^. Animals, including humans, cannot synthesize biotin and must rely on exogenous sources for biotin and thus the enzymes of biotin biosynthesis are attractive drug targets for development of novel antibacterial agents^3, 4^.

**Figure 1:**
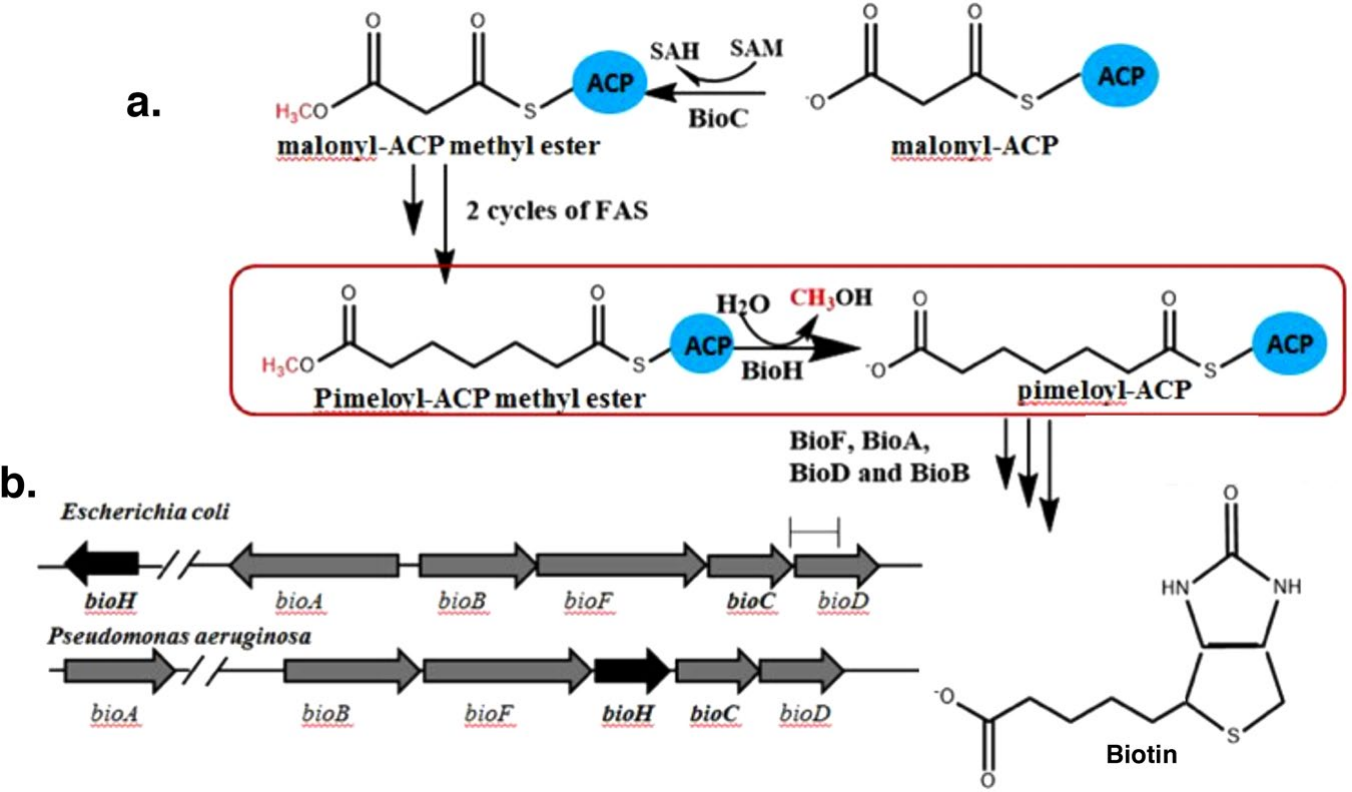
The *E. coli* biotin biosynthesis pathway and genetic organizations of the *E. coli* and *P. aeruginosa* biotin synthesis genes. (**a)** Scheme of the biotin synthesis pathway. (**b)** The biotin synthesis gene organizations of *E. coli* and *P. aeruginosa*. Note that in *E. coli*, the five *bioABFCD* genes are located within the biotin synthesis (*bio*) operon at min 17 of the chromosome map whereas the *bioH* gene is well removed from the *bio* operon at min 74 of the genetic map (note the broken lines).

Biotin functions only when covalently attached to its cognate proteins which are involved in key metabolic carboxylation and decarboxylation reactions. Biotin acts as part of a long “swinging arm” that transfers intermediates between active sites of key metabolic enzymes by covalent substrate channeling^5–7^. Biotin consists of two fused heterocyclic rings plus a valeric acid chain (Fig. 1a)^8^. The biotin synthetic pathway can be readily divided into early and late stages. The enzymes of the late stage, those required for the assembly of the two heterocyclic rings, are strongly conserved across the archea, bacteria, plants and fungi and their biochemistry and structures are well understood^9, 10^. In *E. coli* and many other bacteria these steps are encoded in a gene cluster that often is regulated by BirA, a bifunctional protein that acts both as a biotin-protein ligase and a transcriptional repressor^11, 12^. In contrast the early stage, that responsible for synthesis of the pimelate thioester that contributes to the valeric side chain and the first ring carbons, is quite diverse. The pathway for synthesis of this moiety was demonstrated only recently in *E. coli* and consists of enzymes encoded by the *bioH* and *bioC* genes that allow the fatty acid synthesis pathway to make pimelate, a seven carbon dicarboxylic acid^9, 13^ (Fig. 1a). BioC, a carboxyl methyltransferase, initiates biotin synthesis by methylation of the free carboxyl group of a fraction of the key fatty acid synthetic intermediate, malonyl-ACP and thereby appropriates a small portion of malonyl-acyl carrier protein (ACP) from the type II fatty acid synthesis pathway (Fig. 1a). Methylation of the free carboxyl of malonyl-ACP disguises the substrate and allows its entry into the fatty acid synthesis pathway^9, 13^. When the acyl chain has been elongated to seven carbons, the disguise is (and must be) removed by the BioH pimeloyl-ACP methyl ester esterase^13^. A remaining puzzle in the *E. coli* pathway is that BioC is encoded within the *bioABFCD* operon and is transcriptionally regulated with the other operon genes by the BirA repressor/biotin protein ligase. In contrast the *bioH* gene is encoded at a distant location and is not regulated by BirA (Fig. 1b)^14, 15^. However, in other proteobacteria (e.g., *Pseudomonas* species) the *bioH* gene is found within a putative biotin operon where it is generally located immediately upstream of *bioC* (Fig. 1b)^16^. *E. coli* BioH is known to be a rather promiscuous carboxylesterase in that it hydrolyzes the ester bonds of short and medium acyl chain *p*-nitrophenyl esters^17, 18^ and also cleaves the ethyl, propyl and butyl esters of pimeloyl-ACP^13^. Others have reported that BioH cleaves the methyl ester of dimethylbutyryl-S-methyl mercaptopropionate^19^ and fatty acid methyl esters^20^. Moreover like other esterases BioH catalyzes condensation reactions in organic solvents^21–23^. BioH is also an atypical biotin synthetic enzyme in that in many bacteria it is functionally replaced by other nonorthologous esterases of very different sequence^16^. To date BioG most often replaces BioH although several other nonorthologous BioH substitutes have been described that, unlike BioH and BioG, are restricted to very specific bacterial species. Examples are BioK in cyanobacteria^16^, BioV in *Helicobacter* species^24^ and BioJ in *Francisella* species^25^. This unexpected diversity argues that some of the enzymes that cleave the methyl ester of pimeloyl-ACP methyl ester may have arisen recently and hence may not be attuned to the low demands of biotin synthesis (*E. coli* growth requires only a few hundred biotin molecules per cell). Indeed, *E. coli* BioH is a much better catalyst by orders of magnitude than the later enzymes of the pathway BioA, BioB and BioD^26–29^.

Given this situation we hypothesized that the BioH proteins encoded within an operon (“operon-encoded”) may be better attuned to the later enzymes in the pathway than the “freestanding” BioH proteins. This is because their expression would be regulated at the transcriptional level and hence coordinated with expression of the other enzymes. Moreover, expression might also be coregulated at the translational level because the open reading frames of biotin operon genes often overlap with those of the upstream and downstream consistent with translational coupling^30^. Finally the operon-encoded BioH proteins may have been altered to modulate their activities and increase their specificity relative to the freestanding BioH proteins, those encoded outside operons. To test this hypothesis we determined the relative expression levels, catalytic activities and specificities of two BioH proteins, those encoded by the freestanding *E. coli bioH* and the operon-encoded *bioH* of *Pseudomonas aeruginosa* PAO1.

## Results

### Both the freestanding and operon-encoded BioH proteins have similarly high enzymatic activities

**Figure 2:**
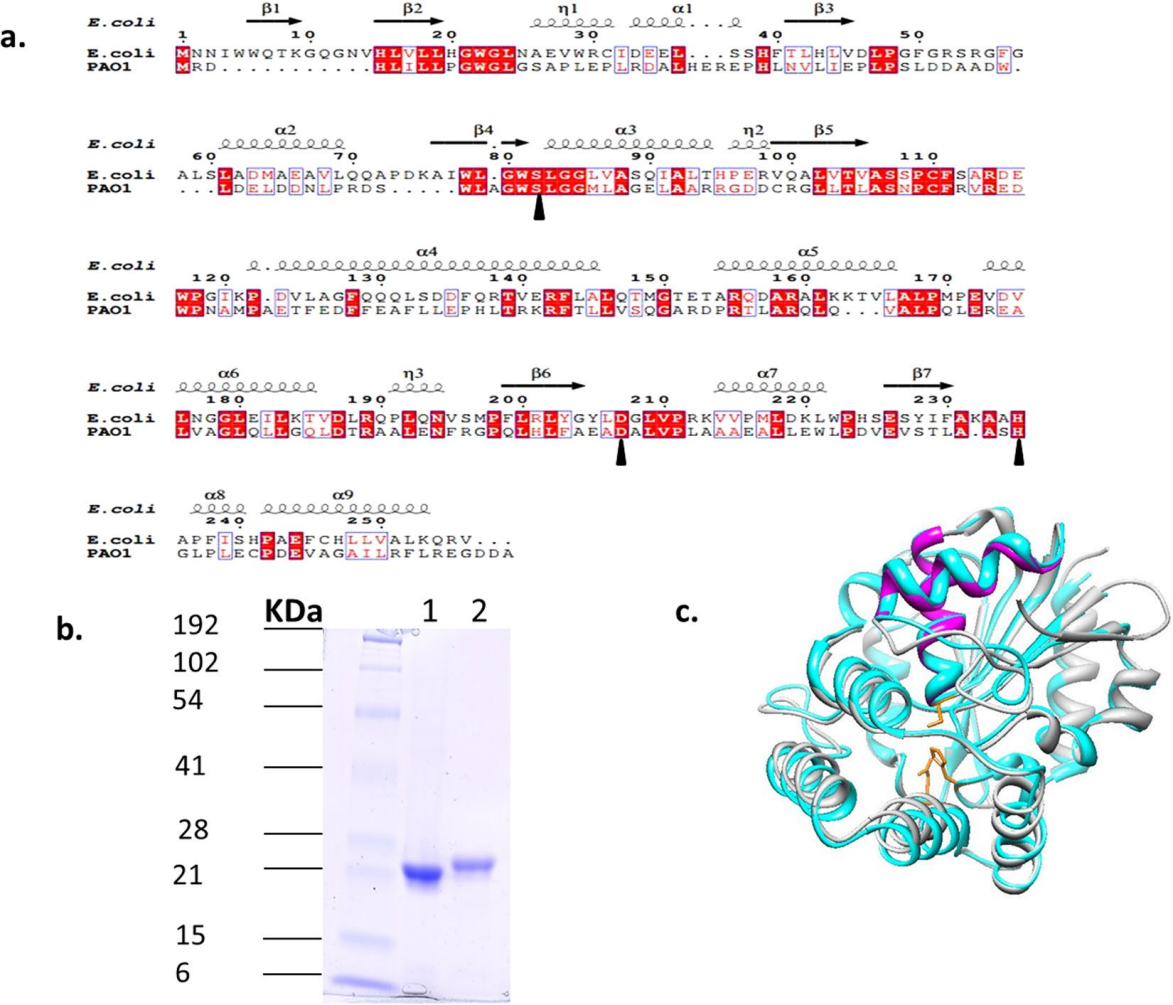
Sequence alignments, purification and modeling of BioH proteins. (A), sequence alignment of the BioH proteins of *E. coli* and *P. aeruginosa* PAO1. (**a)** Conserved residues are shown as white letters on a red background, and similar residues are shown as red letters in blue boxes. The *E. coli* BioH secondary structure (Protein Data Bank ID 1m33) is shown at the top of the panel. The catalytic triad residues are denoted by black arrow heads. (**b)** Purification of P. aeruginosa BioH (lane 1) and *E. coli* BioH (lane 2). The molecular masses of prestained broad-range protein standards (Bio-Rad) are indicated. The proteins were purified as described under Materials and Methods and analyzed by SDS-PAGE on a 15% polyacrylamide gel. (**c)** Structural model of *P. aeruginosa BioH* (cyan) obtained by threading on the structure of *E. coli BioH* (grey) using the SwissModel website and PDB 1m33^17^. Helices 2 and 3 of the lid domain of *E. coli* BioH are coloured magenta. The side chains of the catalytic triad residues are coloured yellow.

The operon-encoded BioH of *P. aeruginosa* has 29% sequence identity to the freestanding *E. coli* BioH over the length of the former (shorter) protein (Fig. 2a). Expression of the *P. aeruginosa* in an *E. coli ∆bioH* strain resulted in robust growth in biotin-free medium as expected from prior studies with various nonorthologous esterases^24, 25, 30, 31^ (data not shown). Alignments of the two BioH proteins argue that the two enzymes share the same esterase catalytic triad (Ser-His-Asp) (Fig. 2a). Indeed, modeling the operon-encoded BioH structures on the known structure of the *E. coli* BioH argues that the structures are almost identical (Fig. 2c). Note that despite its low sequence identity with *E. coli* BioH the *P. aeruginosa* protein is clearly a BioH and not one of the other pimeloyl-ACP methyl ester esterases because those nonorthologous proteins cannot be aligned with *E. coli* BioH even given very permissive alignment parameters^24, 25, 30^.

**Figure 3:**
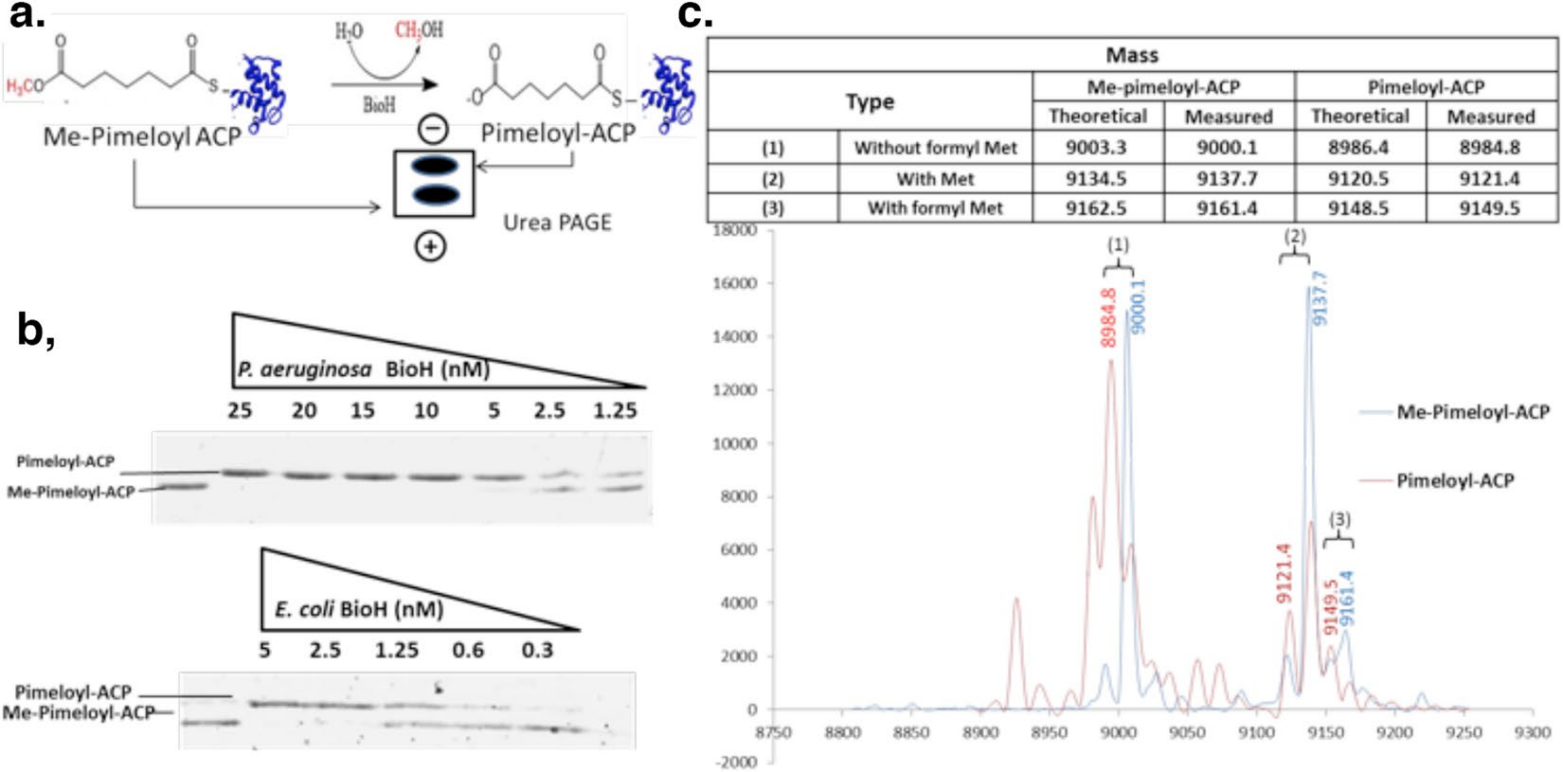
Enzymatic activities of *E. coli* and *P. aeruginosa* BioH proteins against pimeloyl-ACP methyl ester. (**a)** Schematic diagram of the BioH catalyzed reaction and its analysis. (**b)** Enzymatic assays for hydrolysis of pimeloyl-ACP methyl ester to pimeloyl-ACP by *P. aeruginosa* and *E. coli* BioH proteins. The triangles represent BioH levels in a dilution series. (**c)** Mass spectrometric analysis of the BioH reaction. Only the *P. aeruginosa* BioH reaction products are shown. Mass spectra of the products of *E. coli* BioH reactions were reported previously13. Three ACP species were detected when ACP was over-expressed in *E. coli* due to titration of the deformylase and methionine aminopeptidase that process the formylated N-terminal methionine from nascent proteins.

The kinetics of *E. coli* BioH with *p*-nitrophenyl esters of various acyl chain lengths were determined previously^18^. However, *p-*nitrophenyl esters are not physiological BioH substrates and are more easily hydrolyzed than a methyl ester. Therefore, we compared the carboxylesterase activity of the two BioH proteins using the physiological substrate, pimeloyl-ACP methyl ester. N-terminal hexahistidine-tagged versions of *E. coli* BioH and *P. aeruginosa* BioH were readily expressed and purified to homogeneity by nickel-chelate affinity chromatography followed by size exclusion chromatography (Fig. 2b). To test BioH enzymatic activity pimeloyl-ACP methyl ester was synthesized from *E. coli* ACP (Materials and Methods). The pimeloyl-ACP product can be distinguished from the pimeloyl-ACP methyl ester substrate by its slower migration in a conformationally-sensitive urea-PAGE gel system (Fig. 3c)^13, 24, 25, 30, 32^. The reactions were performed with serial dilutions of BioH at a constant substrate concentration (50 μM pimeloyl-ACP methyl ester), production of pimeloyl-ACP by both BioH proteins could be observed with as little as 1.25 nM enzyme (Fig. 3b). For the freestanding *E. coli* BioH, the reaction approached completion with 2.5 nM enzyme whereas the reaction completion was seen at 5 nM for operon-encoded *P. aeruginosa* BioH (Fig. 3b). The *P. aeruginosa* reaction products were analyzed by mass spectrometry which showed conversion of pimeloyl-ACP methyl ester to pimeloyl-ACP by loss of a methyl group (Fig. 3c) as shown previously for *E. coli* BioH^13^. Pimeloyl-ACP methyl ester synthesized from *P. aeruginosa* ACP was also utilized as substrate and gave essentially identical results (data not shown) indicating that the ACP source was immaterial. This was expected since *P. aeruginosa* ACP is 90% identical to *E. coli* ACP and the α2-helices of the two proteins have identical sequences. Since α2-helix provides almost all of the residues that interact with BioH^1^, *E. coli* ACP is an excellent surrogate for *P. aeruginosa* ACP. Note that quantitation of BioH activity is problematical and accurate Michaelis-Menten data could not be obtained. This was because detection of the cleavage reaction requires that an appreciable fraction of the substrate be converted to product (the substrate concentration can go to zero) and densitometry is imprecise because separation quality and background vary from gel to gel. Moreover, substrate concentrations cannot be varied over a wide range because of lack of sensitivity at low concentrations and poor resolution of substrate and product at high concentrations. For these reasons, we have chosen to assay BioH activity by varying the enzyme concentration. Indeed, relative to the freestanding *E. coli* BioH only twice the enzyme concentration was required for the operon-encoded *P. aeruginosa* BioH to complete pimeloyl-ACP methyl ester hydrolysis. Hence, both the freestanding BioH and operon-encoded BioH proteins possess similarly high enzymatic activity.

### The operon-encoded BioH has a broad substrate specificity similar to that of the freestanding BioH

**Figure 4:**
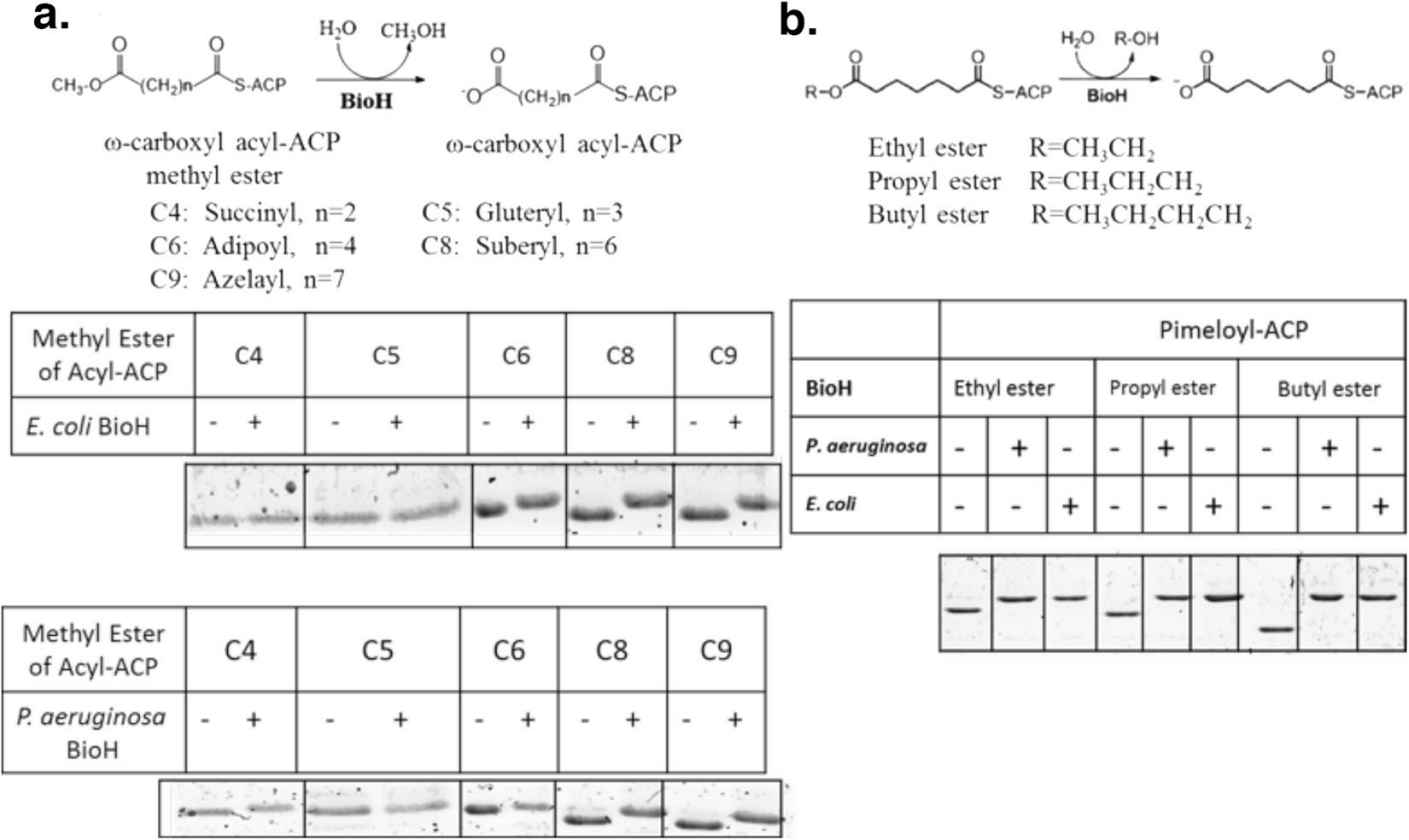
BioH-catalyzed hydrolysis of the ester bonds of various ω-carboxyl acyl-ACP esters. (**a)** The reactions contained 50 mM Tris-HCl (pH 7.0), 5% glycerol, 5 μg/ml BioH and 2 mM pimeloyl-ACP esters and were incubated at 37 °C for 30 min. BioH cleavage of the methyl ester moieties of acyl-ACP methyl esters having acyl chain lengths of 4 to 9 carbon atoms. (**b)** BioH cleavage of the ethyl-, butyl- and propyl- esters of pimeloyl-ACP.

*E. coli* BioH has a small lid domain and a large core domain where the active site resides^1, 18^. The function of the lid domain was unclear until Agarwal and coworkers^1^ showed that the lid domain provides most of the residues that interact with the α2-helix of the substrate ACP moiety consistent with later molecular dynamics simulations of BioH specificity^33^. Previous experiments have shown that the freestanding *E. coli* BioH hydrolyzed the ester bonds of acyl-ACP esters of different chain characteristics *in vitro* ^13^. To test if this is the case for the operon-encoded BioH, we synthesized various ω-carboxyl-acyl-ACP esters from *E. coli* ACP and assayed the *in vitro* abilities of the two BioH proteins to cleave the ester moieties of these substrates. Both BioH enzymes readily hydrolyzed the methyl esters of adipyl-, suberyl- and azelayl-ACPs (the C6, C8 and C9 acyl chains) but failed to cleave succinyl-ACP methyl ester (Fig. 4a). Glutaryl (C5)-ACP methyl ester was cleaved less efficiently than pimeloyl-ACP methyl ester by both BioH proteins (Fig. 4a). In addition, both BioH proteins cleaved the ethyl, propyl and butyl esters of pimeloyl-ACP (Fig. 4b). These results indicate that operon-encoded BioH and freestanding BioH have similarly broad substrate specificities.

### The operon-encoded *bioH* is transcribed at higher levels than the freestanding *bioH*

**Figure 5:**
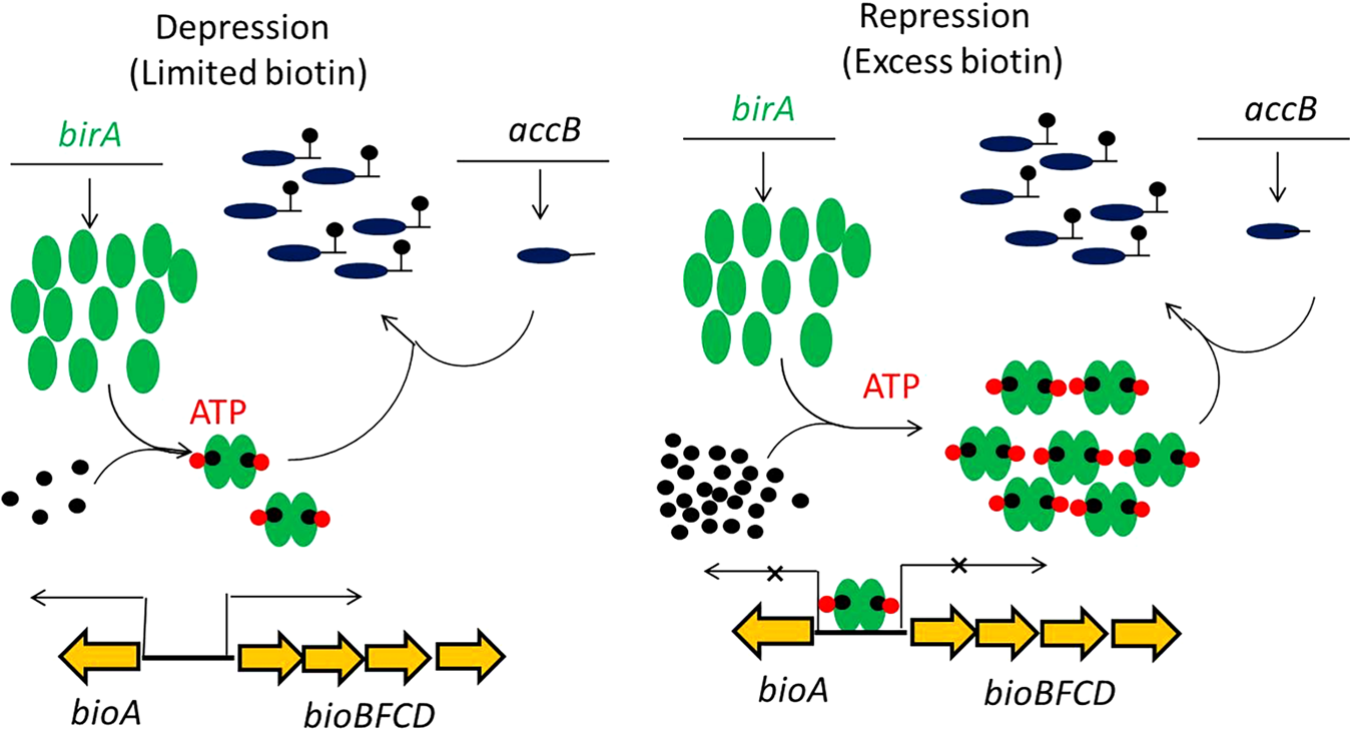
Regulation of the *E. coli* biotin operon transcription by the BirA ligase/biotin repressor. General model of BirA *bio* operon regulation in *E. coli*. Green ovals denote BirA; tailed blue ovals denote the biotin acceptor protein, AccB; black dots denote biotin; black dots with red pentagons denote biotinoyl-5′-adenylate. The left panel shows derepression of *bio* operon transcription engendered by biotin limitation whereas the right panel shows the transcriptionally repressed state under excess biotin.

Biotin operon transcription is regulated in response to biotin availability by BirA^11, 12^. When the intracellular biotin concentration is high and the biotin acceptor proteins have been modified, biotin will be activated by BirA to form the BirA-biotinyl-5′-AMP complex, the ligation reaction intermediate^34^. Bound biotinyl-5′-AMP greatly increases the ability of BirA monomers to form dimers, the species required for binding of the operator located between the divergent *bioA* and *bioBCDF* genes resulting in repression of both transcripts (Fig. 5). When the intracellular biotin concentration is low and unmodified biotin acceptor protein concentration is present, BirA will transfer biotin from biotinyl-5′-AMP to the specific lysine residues of its cognate proteins. The consumption of biotinyl-5′-AMP results in loss of BirA dimers and derepression of biotin operon transcription (Fig. 5)^14, 34, 35^.

**Figure 6:**
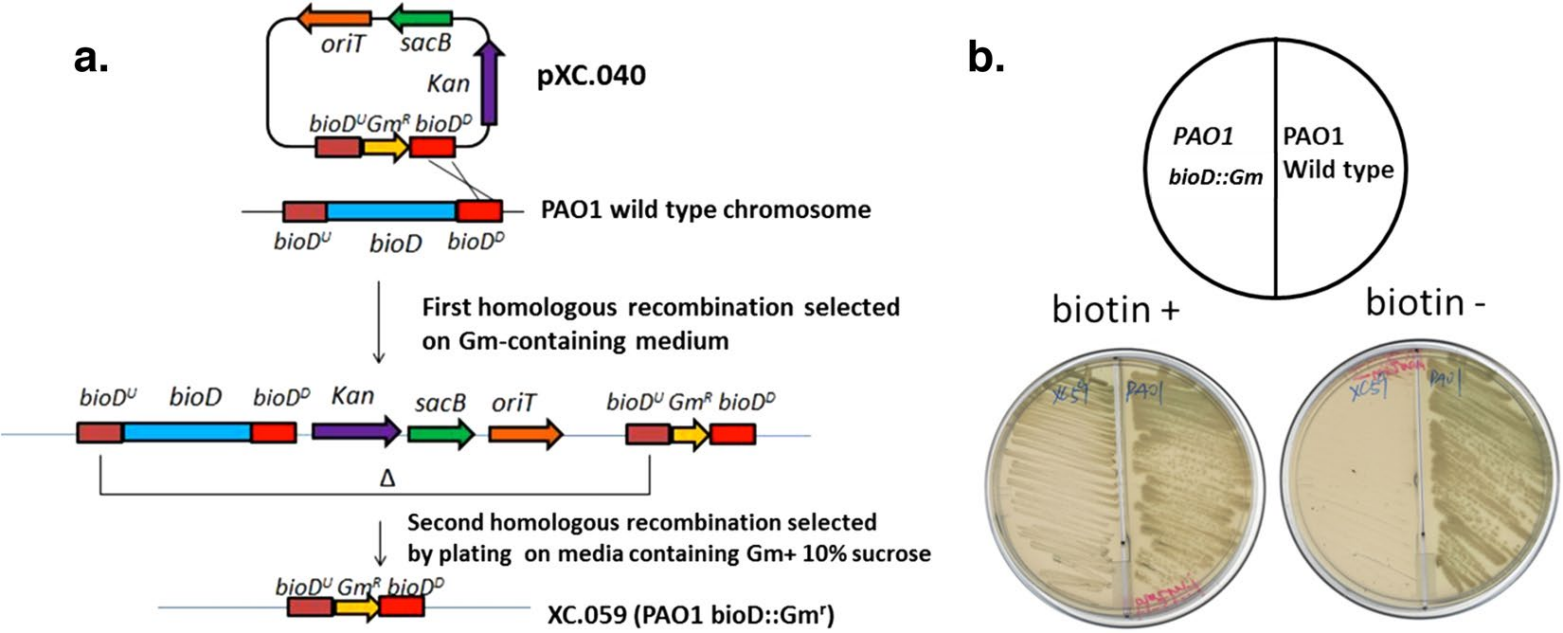
Construction and characterization of the *P. aeruginosa bioD::Gm* strain. (**a)** Recombination events required to generate the mutant strain by *sacB* counterselection gene replacement. (**b)** Characterization of the mutation by biotin complementation. Strain XC.059 was streaked on M9 defined medium plates containing 40 nM biotin (left plate) or lacking biotin (right plate) Abbreviations: kan, kanamycin-resistant gene; Gm^R^, Gm resistant marker; ori, pMB1 origin of replication; *oriT*, origin of transfer; *sacB*, levansucrase-encoding gene.

BioC and BioH work in tandem to synthesize the pimeloyl-ACP precursor that provides the biotin valeric acid chain and upon reaction with alanine begins biotin ring synthesis^13, 36^. In order to compare *bioH* and *bioC* transcription levels in *E. coli* and *P. aeruginosa*, a *bioD::Gm* biotin auxotrophic strain of *P. aeruginosa* was constructed (Fig. 6a). This strain (XC.059) grew on defined medium plates only upon supplementation with biotin indicating successful disruption of *bioD* (Fig. 6b).

Real-time qRT-PC was performed to examine the transcription levels of *bioH* versus *bioC* in the *E. coli ∆bioD* strain STL111 and *P. aeruginosa bioD::Gm* strain. The cultures were grown in defined medium containing 1.6 nM biotin, a limiting concentration which permits growth of biotin auxotrophs and derepression of biotin operon transcription or in 40 μM biotin, a concentration that represses expression. The cells of mid-log phase cultures of *E. coli ∆bioD* and *P. aeruginosa bioD::Gm* strains grown in defined medium containing either of the two biotin concentrations were collected and used to obtain total RNA preparations. The real-time qPCR-based analyses of transcriptional profile showed that in *the E. coli ∆bioD* strain, *bioC* gene was transcribed about 3-fold more than the *bioH* gene under derepression conditions (1.6 nM biotin), whereas the transcription levels of *bioC* and *bioH* expression were similar under repressed conditions (40 μM biotin) (Fig. S1a). The relative expression levels of *bioH* and *bioC* in *the P. aeruginosa bioD::Gm* strain remained the same under both biotin concentrations (Fig. S1b) consistent with cotranscription of the genes. The real-time qRT-PCR data indicates that the freestanding *bioH* gene is transcribed at lower levels than the operon-encoded *bioH* gene.

### Freestanding BioH is produced at lower levels than operon-encoded BioH

**Figure 7:**
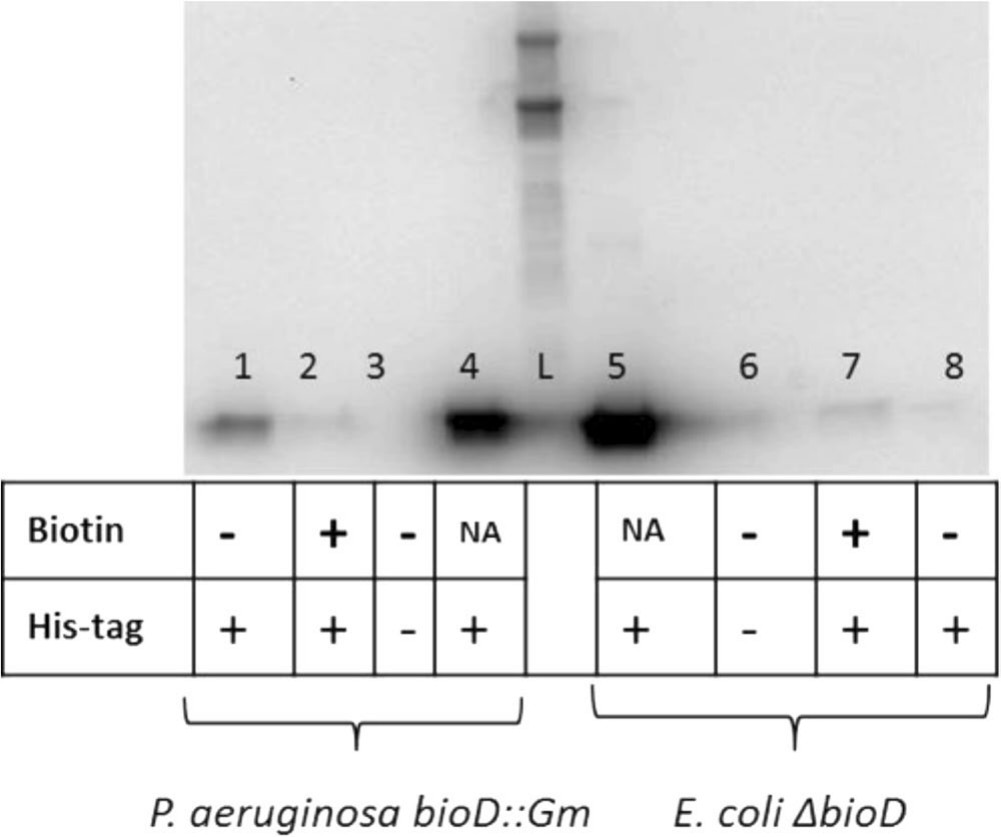
Western blot analyses of freestanding and operon-encoded BioH production. Equal volumes of the freestanding and operon-encoded protein eluates were loaded into each lane of an SDS-polyacrylamide gel. After electrophoresis the proteins were transferred to Immobilon-P and the membranes were subjected to immunoblotting with anti-His_6_ tag antibody. Lane 1, the *P. aeruginosa bioD::Gm* strain expressing chromosomal His_6_-tagged BioH under limited biotin conditions (1.6 nM); Lane 2, the *P. aeruginosa bioD::Gm* strain expressing chromosomal His_6_-tagged operon-encoded BioH under excess biotin conditions (40 μM); Lane 3, negative control, *P. aeruginosa bioD::Gm* strain expressing operon-encoded native BioH lacking a His_6_ tag under limited biotin conditions (1.6 nM); Lane 4, positive control, purified *P. aeruginosa* BioH protein with a C-terminal His_6_-tag; L, ladder; Lane 5, positive control, purified *E. coli* BioH protein with a C-terminal His_6_-tag; Lane 6, negative control, the *E. coli ∆bioD* strain expressing the native freestanding chromosomal BioH lacking a His_6_ tag under limited biotin conditions (1.6 nM); Lane 7, the *E. coli ∆bioD* strain expressing a chromosomal His_6_-tagged *bioH* under excess biotin (40 μM) conditions and Lane 8, the *E. coli ∆bioD* expressing the chromosomal His_6_-tagged BioH under limited biotin conditions (1.6 nM).

In order to detect the levels of the freestanding and operon-encoded BioH proteins, we modified the chromosomal *bioH* genes of both bacteria with a sequence encoding a His_6_ C-terminal extension to allow antibody detection. Cells were collected at mid-log phase from cultures grown in defined medium that contained either excess or limited biotin. Note that biotin biosynthetic enzymes are expressed at very low levels because the demand for biotin is miniscule. Growth of *E. coli* requires only a few hundred biotin molecules per cell^37^. Consistent with the low demand for biotin ribosome profiling indicates that an *E. coli* cultures grown on defined medium contain only 158 molecules of BioH per cell^38^. Indeed, we were unable to detect *E*. *coli* and *P*. *aeruginosa* His_6_-tagged BioH proteins in unfractionated cell extracts by western blot analysis. For this reason we enriched and concentrated the BioH proteins from 500 ml cultures by binding to a nickel chelate column prior to western blot analysis. Equal amounts of protein from each culture were loaded on the columns and then equal volumes of the eluted enriched preparations were loaded on the denaturing electrophoresis gels together with purified *E. coli* and *P. aeruginosa* His_6_-tagged BioH proteins as positive controls. Upon visualization with an anti-hexahistidine antibody we observed different levels of BioH production in *E. coli ∆bioD* and *P. aeruginosa bioD::Gm* strains (Fig. 7). Under biotin-limited conditions, a BioH protein band was readily observed in the *P. aeruginosa bioD::Gm His*_*6*_ *-bioH* strain whereas in cells grown with excess biotin, no BioH band was detectable indicating repression by biotin (Fig. 7). In contrast in the *E. coli ∆bioD His*_*6*_ *-bioH* strain the BioH protein band was very faint even in biotin-limited cells consistent with its reported lack of regulation by biotin availability^14, 15^ (Fig. 7). Given these data and the real-time qRT-PCR data it seems that the freestanding *bioH* mRNA is translated markedly less efficiently than the operon-encoded *bioH* mRNA. The efficient translation of the *P. aeruginosa* mRNA might result from translational coupling with the upstream *bioF* gene.

### Bioinformatic analysis of freestanding BioH and operon-encoded BioH proteins

**Figure 8:**
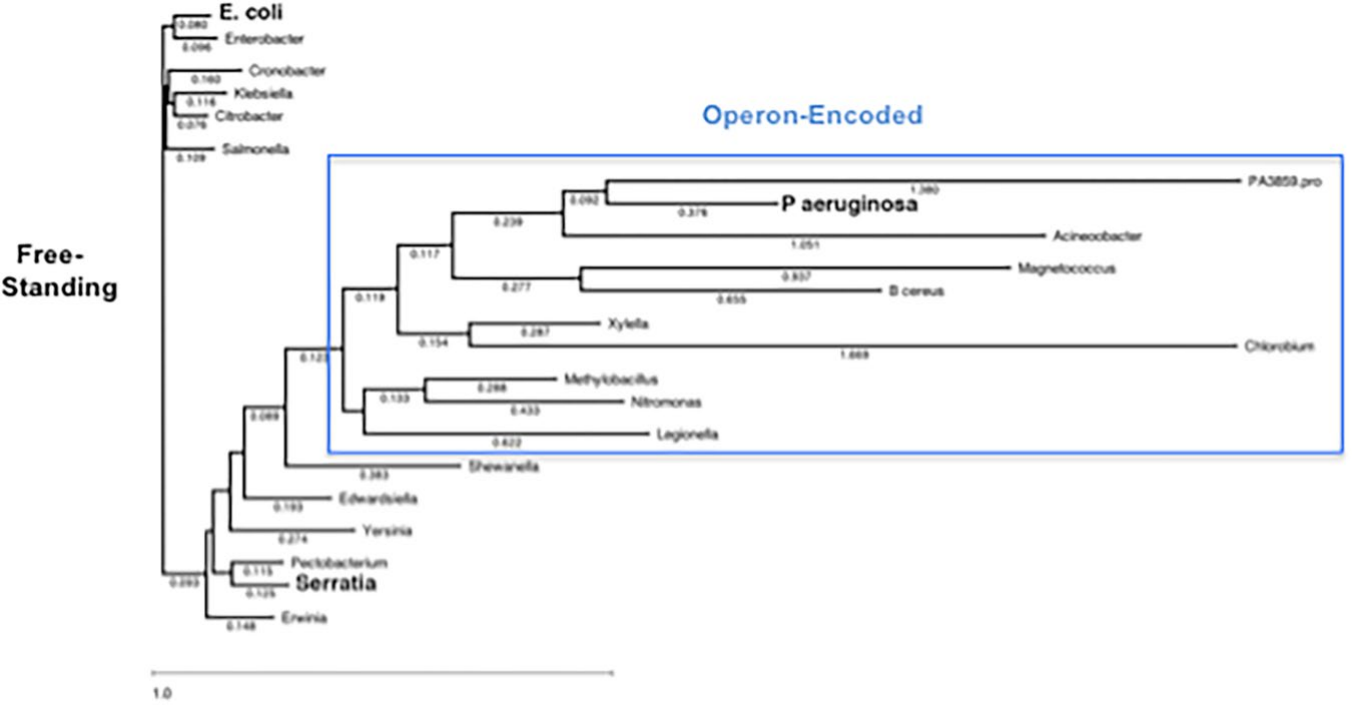
Phylogenetic tree of bacterial BioH proteins. The three BioH proteins with demonstrated pimeloyl-ACP methyl ester cleavage activity are given in large bold type. The PA3859 protein is included as the outlier since it is a *P. aeruginosa* α/β-hydrolase known to lack pimeloyl-ACP methyl ester cleavage activity^31^. The blue dotted box with the exception of PA3859 denotes the operon-encoded BioH proteins. The other BioH proteins (those outside the blue box) are defined as freestanding. The six bacterial species at the top left have very similar genomes with *bioH* immediately adjacent to the divergently transcribed *gntX* gene. The other freestanding *bioH* genes were identified using the adjacent divergently transcribed *comFC* gene. The additional criteria used in identification of all freestanding genes are described in the text. The numbers denote genetic distances which are estimates of the degree of divergence between two sequences and indicates the number of mutations (amino acid residue changes or insertion-deletions) that have occurred since the two sequences shared a common ancestor. The tree was calculated using Clustal Omega (http://clustal.org/omega/) and the Kimura metric^39, 40^. B. cereus denotes *Bacillus cereus* and closely related species because other bacilli (e.g., *Bacillus subtilis*) synthesize biotin using a pathway that does not utilize BioH and BioC.

A phylogenetic tree was constructed using Clustal Omega followed by analysis according to Kimura^39, 40^. The phylogeny of freestanding BioH from *E. coli* and operon-encoded BioH from *P. aeruginosa* were determined together with other BioH homologs (Fig. 8). As discussed below this analysis revealed that the freestanding BioH proteins and operon-encoded BioH proteins form distinct clades.

## Discussion

BioH is a member of the α/β-hydrolase fold superfamily, a protein family which is readily evolvable to give enzymes of diverse function^41^. The BioH function seems something of a “wild card” among biotin synthetic enzymes since various nonorthologous substitutes of *bioH* have been observed in numerous bacterial genomes. In place of *bioH*, genes called *bioG*, *bioK*, *bioJ* and *bioV* and the proteins they encode have been shown to have *bioH* function *in vivo* and *in vitro* ^16, 24, 25, 30^. BioH functions in tandem with BioC^13, 36^. The *bioC* gene is conserved among highly divergent groups of bacteria, even in those where *bioH* has been displaced by genes encoding proteins having very low sequence identity with BioH. *E. coli* BioH, the canonical freestanding enzyme, has a broad substrate specificity and modestly higher enzymatic activity than the *P. aeruginosa* operon-encoded BioH. However our hypothesis to explain the ambiguity between freestanding *bioH* and operon-encoded *bioH* genes has now been resolved by the demonstration that the two BioH proteins have similar enzymatic activities and similar broad substrate specificities. Hence, the expectation that expression of the operon-encoded *bioH* would have become “domesticated” and perhaps would be transcribed and translated at lower levels than the freestanding *bioH* was not fulfilled. Indeed, the operon-encoded enzyme was expressed at higher levels.

Unexpectedly both the freestanding BioH and operon-encoded BioH proteins have much higher enzymatic activities than the *E. coli* biotin synthesis enzymes involved in assembling the heterocyclic rings. In this regard it is interesting that evolution of a *P. aeruginosa* carboxylesterase (PA3859) of unknown function to gain BioH activity required only simple amino acid substitutions^31^. Moreover only when high *in vitro* activity against pimeloyl-ACP methyl ester was attained did the mutant PA3859 proteins support growth at rates similar to that of *E. coli* BioH^31^. A recent study of the enzyme encoded by *Haemophilus influenzae bioG*, the gene most often found in place of *bioH* immediately upstream of *bioC*, encodes an enzyme of activity comparable to that of *E. coli* BioH^42^. Therefore, it is tempting to conclude that BioH and its related isofunctional enzymes all have high enzymatic activity and might have been captured for evolutionary contingency by natural selection^43^. This, together with our results, suggests that homologous proteins that have diverged extensively while retaining the required enzymatic activity can provide both freestanding BioH and operon-encoded BioH function. High activity would have the advantage of preventing wasteful elongation of pimeloyl-ACP methyl ester to longer chain length species. However, extremely high esterase activity could be counterproductive because glutaryl-ACP methyl ester might be cleaved which would short circuit pimelate synthesis.

The diversity of the esterases that cleave pimeloyl-ACP methyl ester is atypical and intriguing. Generally speaking when a pathway is conserved, all of the enzymes are conserved. Our finding that the freestanding BioH proteins form a clade that seems distinct from the operon-encoded encoded BioH proteins adds to the intrigue (Fig. 8). The freestanding *bioH* genes seem to be more recent acquisitions than the esterases encoded within biotin operons because the coding sequences of the operon-encoded *bioH* genes often overlap with those of the upstream and downstream genes (8 bp each for the *P. aeruginosa bioH*). The development of this sophisticated arrangement necessarily involves making a coding region from noncoding DNA, a process that seems likely to require numerous cycles of purifying selection and optimization^44^.

Many genes encoding putative α/β-hydrolases are present in bacterial genomes and thus freestanding pimeloyl-ACP methyl ester hydrolases cannot be recognized unless the proteins align very well with a protein of known activity or lie in a specific genome neighborhood. Indeed, both *E. coli bioH* and *F. novicida bioJ* were discovered by serendipity in searches for mutants in pathways unrelated to biotin synthesis whereas *bioV* was isolated by complementation screening of a library of *H. pylori* genome fragments. Searches for transcriptional regulatory sequences upstream of coding sequences is also problematical since expression of the known freestanding genes (*E. coli bioH* and *F. novicida bioJ*) are not subject to the same regulation as the cognate biotin operons (the *H. pylori* biotin synthetic genes are scattered about the genome). Hence numerous unrecognized freestanding pimeloyl-ACP methyl ester hydrolases may be present in bacterial genomes. However, Akatsuka and coworkers reported a genome neighborhood^45^ that seems to provide a means to identify some freestanding *bioH* genes. They observed that an ORF upstream of the *Serratia bioH* encoded a protein with homology to *Haemophilus influenzae* ComFC and that this genome neighborhood was conserved in several other bacterial species^45^. Moreover in a few bacteria other biotin synthetic genes are found neighboring *bioH* and *comFC*. These workers clearly demonstrated that the putative *Serratia* gene encoded a *bona fide* BioH. The protein is 70% identical to *E. coli* BioH and expression of the gene complemented growth of an *E. coli bioH* mutant strain. Moreover, disruption of the gene engendered a biotin requirement^45^. Hence, the linkage to *comFC* seems a reliable indication that the downstream gene encodes a BioH homologue rather than another type of α/β-hydrolase. If so, the encoded protein should show high sequence identity with the *E. coli* and *Serratia* BioH proteins and the biotin operons of candidate bacteria should lack an α/β-hydrolase-encoding gene. We found that these criteria were fulfilled in several diverse bacterial species: *Yersinia, Erwinia, Pectobacterium, Enterobacter, Klebsiella, Cronobacter, Citrobacter, Klebsiella, Salmonella, Shewanella* and *Edwardsiella*. All these genomes encoded a protein having greater than 63% identity to the *E. coli* and *Serratia* BioH proteins (except *Shewanella* which was 45%) and several approach 80% identity. In contrast two operon*-*encoded BioH proteins, those of *P. aeruginosa* and *Bacillus cereus*, showed much lower identities with the freestanding *E. coli* BioH protein, 29% and 18%, respectively. Moreover the *P. aeruginosa* and *Bacillus cereus* proteins showed only 21% identity. Indeed when the BioH sequences were submitted to Clustal Omega alignment and the results analyzed according to Kimura^31, 39, 40^, the freestanding and operon-encoded BioH proteins fell cleanly into two clades. Moreover, the same clades were found when two other analysis approaches, Uncorrected Pairwise Distance and Scoredist^46^ were applied.

The bioinformatics analyses (Fig. 8) argue that the freestanding BioH proteins have diverged less from the common ancestor than the operon-encoded proteins. This may be the case but the situation is greatly complicated by the presence of nonorthologous genes that encode both freestanding and operon-encoded proteins having pimeloyl-ACP methyl ester hydrolase activity^16, 30^. Another bewildering conundrum is the species specificity of the nonorthologous BioJ, BioK and BioV proteins. An especially puzzling case arises in *Francisella* species which have a *bioABFCD* operon that has the same gene order and spacing as that of *E. coli*. Moreover, the *Francisella* BirA regulatory protein weakly regulates transcription of the *E. coli bio* operon^47^. However, *Francisella* species lack BioH and instead encode BioJ, an appreciably larger freestanding α/β-hydrolase that lacks sequence similarity with BioH^25^. Given these considerations we are unable to propose a straightforward phylogeny for BioH.

## Materials and Methods

### Chemicals, Bacterial Strains and Growth Media

The antibiotics and most chemicals used in this study were purchased from Sigma or Thermo Fisher unless noted otherwise. PCR amplification was performed using *Pfu* (Stratagene) or *Taq* (New England Biolabs) polymerases. New England Biolabs supplied restriction enzymes and T4 DNA ligase. DNA sequencing was performed by AGCT, Inc. Invitrogen provided the Ni^++^-agarose column. *P. aeruginosa* PAO1 genomic DNA was extracted using a genomic DNA purification kit (Promega). Antibiotics were used at the following concentrations (in μg·mL^−1^): kanamycin sulfate, 30; chloramphenicol; gentamycin sulfate 50 and tetracycline hydrochloride, 60. The bacterial strains used were derivatives of *E. coli* K-12 or *P. aeruginosa* PAO1 (Table S1). The rich medium used for strain growth was LB broth. The defined medium was M9 medium supplemented with 0.3% (wt/vol) glucose and 0.1% (wt/vol) Casamino acids.

### Plasmid constructions

The *P. aeruginosa bioH* and *bioC* genes were amplified from *P. aeruginosa* PAO1 genomic DNA using primers BioH-F, BioH-R and BioC-F, BioC-R (Table S2) and ligated into pET-28b(+) (Novagen) between the NcoI and XhoI sites for expression of the protein having a C-terminal hexahistidine tag. Plasmid pXC.039 (Table S1) encoding the *P. aeruginosa* C-terminal His-tagged BioH was sequence verified and transformed into BL21(DE3) to give strain XC.037. Plasmid pSTL6 encoding the *E. coli* BioH with a C-terminal hexahistidine tag and pSTL42 encoding the *E. coli* BioH and a C-terminal hexahistidine-tagged BioC were from laboratory stocks.

### Protein Purification

Strain XC.037 carrying pXC.039 encoding *P. aeruginosa* BioH and strain STL14 carrying pSTL6 encoding *E. coli* BioH (Table S1) were grown to OD600 of 0.8 in LB-kanamycin medium at 37 °C followed by induction for 4 h by addition of 1 mM IPTG. The cells of a 500 ml culture of each strain were collected. All protein purification and manipulations were performed at 4 °C or on ice. The cell pellets were suspended in lysis buffer containing 20 mM sodium MOPS (pH 8.0), 500 mM NaCl, and 10% glycerol and lysed by multiple passages through a French Press. The soluble cell extract was collected and mixed with Ni-NTA resin (Qiagen) for 2 h. The resin was then loaded into a column and washed twice with 40 mM lysis buffer containing 30 mM imidazole. The column was eluted with 250 mM imidazole and protein fractions were collected. Protein purification was monitored by SDS/PAGE. The concentrated protein solutions were dialyzed overnight in dialysis buffer containing 25 mM sodium MOPS, 10% glycerol, 1 mM tris(2-carboxyethyl)phosphine hydrochloride and 0.2 M NaCl (pH 7.5) followed by flash freezing and storage at −80 °C.

### Structural Modeling and Sequence Alignment

A model of *P. aeruginosa* PAO1 BioH was determined by threading it with the *E. coli* BioH crystal structure (PDB: 1m33) using the automated mode of SWISS-MODE^48–50^. The final image was generated using the UCSF Chimera package^51^. Sequence alignment was conducted using ClustalW2, and the final output shown in Fig. 2 was created by ESPript 3.0^52^.

### Protein mass spectrometry

BioH reaction mixtures (20 μl) were loaded onto Vivapure D Mini H columns (Sartorius Stedim) which were washed twice with loading buffer (25 mM sodium MES, 10 mM DTT, pH 6.1) containing 250 mM LiCl. Acyl-ACPs were eluted with same buffer containing 500 mM LiCl followed by dialysis against 200 mM ammonium acetate overnight at 4 °C using a 3,500 molecular weight cut-off membrane. The samples were dried under a nitrogen stream^53^. Mass spectral analyses were performed by the University of Illinois Mass Spectrometry Laboratory. The mass spectra were collected under low resolution in positive ion mode on an UltrafleXtreme MALDI TOF/TOF mass spectrometer (Bruker Daltonics, Bremen, Germany) equipped with a frequency tripled Nd–YAG solid state laser using the FlexControl 1.4 software package (Bruker Daltonics).

### Enzyme Activity Assays

The *E. coli* and *P. aeruginosa* ACP proteins were expressed and purified as previously described^32^. Enzymatic assays of the BioH proteins were performed using the protocol established for *E. coli* BioH with modifications^1^. Each reaction contained 50 mM Tris-HCl (pH 7.0), 5% glycerol, 50 μM acyl-ACP esters and a series of BioH dilutions (0.3–25 nM). The total volume for each reaction was 5 μL. A premix of buffer and pimeloyl-ACP methyl ester (or a shorter or longer homologue) was incubated at 37 °C for 1 min without BioH. Each reaction was initiated by adding BioH and incubated at 37 °C for 2 min. The reactions were stopped by adding an equal volume of 10 M urea and placed on dry ice. For analysis the reaction mixtures were loaded into 20% PAGE gels containing 2.5 M urea and run at 130 V for 2.5 h. Acyl-ACP esters with different chain lengths and ester moieties were synthesized as previously described^1^ ^{Lin, 2010 #65}^.

### Construction of the *P. aeruginosa bioD::Gm* mutant strain

The *P. aeruginosa bioD:: Gm* mutant strain was constructed by following the procedure described by Lei *et al*.^54^. Briefly, to disrupt *P. aeruginosa bioD*, a suicide plasmid was constructed as follows. The 150 bp regions upstream and downstream of *bioD* (called UpbioD and DownbioD, respectively) were amplified with *Pfu* DNA polymerase using strain PAO1 genomic DNA as the template and either oXC48 and oXC51 (for UpbioD) or oXC49 and oXC50 (for DownbioD) as the primers (Table S2). Primers oXC50 and oXC51 both added BamHI sites in the end. A 750 bp gentamicin resistance cassette was cut by restriction enzyme BamHI from plasmid p34s-Gm^55^. The PCR products of UpbioD, DownbioD and DNA fragment of Gm were purified, and overlapping PCR was carried out using primers oXC48 and oXC49, which added EcoRI and HindIII restriction sites. The 1,000 bp *∆PabioD::Gm* PCR fragment was cloned into the same sites of pK19mobsacB to yield pXC.040. Plasmid pXC.040 was then transformed into the *E. coli* donor/helper strain S17.1 and transferred by conjugation into *P. aeruginosa* PAO1. The cells were spread on LB agar plates containing chloramphenicol (to select against the donor strain) plus gentamicin to select for integration of the nonreplicating plasmid into the chromosome of the recipient. Colonies resistant to chloramphenicol and gentamicin were then counter-selected on 10% sucrose LB agar plates. Colonies were screened by colony PCR using primers oXC48 and oXC49 (Table S2).

### Modification of genomic *bioH* genes with a hexahistidine (His_6_) coding sequence

Insertion of a sequence encoding C-terminal His_6_ tag into the *E. coli* genomic *bioH* was performed as by a standard method^56^. Strain STL111 (MG1655 *∆bioD::kan*) was used as the target strain. Briefly, the 35 bp N-terminal and C-terminal regions of *bioH* were amplified with *Pfu* DNA polymerase using pKD3 as the template and P1-P2 as the primers (Table S2). The sequence encoding a His_6_ tag and a stop codon were included on the P1 priming site. The resulting 1.1-kbp PCR product was purified, treated with DpnI and transformed into strain XC.047 carrying the Red recombinase plasmid pKD46 (Table S1). Colonies that grew on LB chloramphenicol plates indicated that the double homologous recombination was successful and the sequence encoding the His_6_ tag had been inserted onto the end of the *bioH* gene. Finally, the chloramphenicol marker was excised by the Flp recombinase encoded by pCP20 (Table S1) to yield XC.052. The genomic His_6_ tagged *bioH* was then PCR amplified with primers oXC53 and oXC54 and sequence verified.

Insertion of a sequence encoding a C-terminal His_6_ tag into the *P. aeruginosa* genomic *bioH* was performed by the counter-selection method described above. Strain XC.059 (PAO1 *bioD::Gm*) was used as the target strain. In brief, the 2.6 kb *bioH::His*_*6*_-Tet PCR fragment was inserted into the same sites of pK19mobsacB to yield pXC.041. Plasmid pXC.041 was then transformed into the *E. coli* donor/helper strain S17.1 and transferred by conjugation into XC.059 (PAO1 *bioD::Gm*). Cells were spread on LB agar plates containing chloramphenicol (to select against the donor strain) plus tetracycline (60 μg/ml) to select for integration of the nonreplicating plasmid into the chromosome of the recipient. Colonies resistant to both chloramphenicol and tetracycline were then counter-selected on 10% sucrose LB plates. Colonies that grew indicated that the double homologous recombination was successful and the sequence encoding the His_6_ tag had been inserted onto the end of the *P. aeruginosa bioH* gene. The insertion was verified by sequencing the PCR fragment obtained using primers oXC126 and oXC131 (Table S2). The verified strain was named XC.109.

### RNA isolation and real-time quantitative RT-PCR

Mid-log phase cultures of *E. coli ∆bioD* (STL111) and *P. aeruginosa bioD:: Gm* (XC.059) grown in M9 medium supplemented with low (1.6 nM) and high (40 μM) biotin concentration were collected for total bacterial RNA preparations. The RNeasy bacterial RNA isolation kit (Qiagen, Hilden, Germany) was utilized. The quality of the acquired RNA samples was visualized using agarose gel electrophoresis.

Real time RT-PCR reaction system (20 μL) contained 12.5 μL of iQTM SYBR Green Supermix, 1 μL of each primer, 1 μL of the diluted cDNA sample, and 4.5 μL of sterile water. All data were collected in triplicate on a Mastercycler eprealplex2 (Eppendorf), using the program of a denaturing cycle at 95 °C for 2 min, 40 cycles comprising 95 °C for 15 sec, 60 °C for 15 sec, and 68 °C for 20 sec and a final step featuring with gradient temperature from 60 °C to 90 °C for dissociating double stranded DNA products. Amplification efficiency for all primer pairs was evaluated using serial ten-fold dilutions of pooled cDNA (100, 10, 1, 0.1, 0.01 ng) or plasmids (2500, 250, 25, 2.5, 0.25 fM). Plasmids pSTL42 encoding *E. coli* BioH and BioC, pXC.039 encoding *P. aeruginosa* BioH and pXC.042 encoding P*. aeruginosa* BioC (Table S1) were adjusted to 25 nM (equal to about 100 ng/µl) to give standard templates. To preclude inaccurate DNA concentrations an equal volume mixture of three plasmids was utilized as the 25 nM standard plasmid samples. A primer pair (oXC201, oXC202, Table S2) for the pET plasmid was designed to determine accurate concentrations of each plasmid based upon the 25 nM standard plasmid sample. Real time qRT-PCR was performed for the ten-fold dilutions of the standard plasmid samples (10^−4^ to 10^−8^), and the linear conversion equations between Ct value and template concentration (logarithm) was generated. After five serial ten-fold dilutions, the Ct value of each plasmid was determined and the concentration of each plasmid was calculated based on the “Ct value-concentration” equation generated above. The same method was utilized to determine the concentrations of *E. coli ∆bioD* cDNA samples and *P. aeruginosa bioD:: Gm* cDNA samples using the respective 16 s gene primer pairs (oXC116 & oXC117 and oXC188 & oXC189, Table S2).

Similarly, for generating the “Ct value-concentration” equation for *bioC* and *bioH* genes, qRT-PCR was performed using a serial dilutions (10^−4^ to 10^−8^) of plasmids pSTL42, pXC.039 and pXC.042 as the templates and corresponding primers (oXC92 and oXC93 for *P. aeruginosa bioC*, oXC184 and oXC185 for *P. aeruginosa bioH*, oXC124 and oXC125 for *E. coli bioC*, oXC118 and oXC119 for *E. coli bioH*) to amplify the *bioC* and *bioH* genes of *E. coli* and P*. aeruginosa*, respectively. Finally, the *bioC* and *bioH* copy numbers of *E. coli* cDNA and *P. aeruginosa* cDNA were calculated via the linear equation generated above (1 mol = 6 × 10^23^ copy numbers).

### Western Blot Analysis

Strains XC.052 and XC.109 were grown to mid-log phase in 500 ml of M9 minimum medium plus 0.3% (wt/vol) glucose, 0.1% (wt/vol) Casamino acids with limited (1.6 nM) or excess (40 μM) biotin. The soluble cell extracts were collected and equal amounts of protein were loaded onto a nickel affinity chromatography column. The proteins were purified as above and were analyzed by SDS-PAGE. Equal volumes of soluble proteins were loaded and separated on a 12% SDS-polyacrylamide gel and transferred by electrophoresis to Immobilon-P membranes (Millipore) for 15 min at 15 V. The membranes were preblocked with TBS buffer (100 mM Tris base and 0.9% NaCl, pH 7.5) containing 0.1% Tween 20 and 5% nonfat milk powder. The membranes were probed for 1 h with an anti-His_6_ protein primary antibody (ThermoFisher Scientific) diluted 1:2,000 in the above buffer. Following incubation with a peroxidase labeled anti-mouse secondary antibody (diluted 1:5000; GE Healthcare Life Sciences), the labeled proteins (His_6_-tagged BioH) were detected using Quantity One software.

## Electronic supplementary material

Supplementary Information
