# Supplementary material for: Expression and Activity of the BioH Esterase of Biotin Synthesis is Independent of Genome Context
Source: Sci Rep. 2017 May 19;7:2141. doi: 10.1038/s41598-017-01490-0 (PMC5438404; doi:10.1038/s41598-017-01490-0)
Supplement: Supplementary file 1 — Supplementary Information [file 41598_2017_1490_MOESM1_ESM.pdf]

Supplement to:

Expression and Activity of the BioH Esterase of Biotin Synthesis is Independent of Genome Context

Xinyun Cao, Lei Zhu, Zhe Hu and John E. Cronan

Table S1. Bacterial strains and plasmids

**Strains**	**Relevant Genotype or Description**	**Ref. or Derivation**
E. coli		
MG1655	*E. coli* K-12 wild type	Lab stock
BL21(DE3)	*E. coli* B *ompT hsdSB gal dcm* (DE3)	Lab stock
S17.1	F^- *thi pro hsd*R [RP4-2 Tc::Mu Km:: Tn]	Lab stock
STL14	BL21 (DE3)/pSTL6	[1]
STL111	MG1655 Δ*bioD::kan*^{r}	Lab stock
XC.037	BL21 (DE3)/pXC.039	This study
XC.047	STL111/pKD46	This study
XC.052	MG1655 Δ*bioD bioH::*His_6-FRT	This study
P. aeruginosa		
PAO1		Lab stock
XC.059	PAO1 *bioD::Gm*	This study
XC.109	PAO1 *bioD::Gm bioH::*His_6*-Tet*	This study
**Plasmids**		
pET28b	T7 promoter expression vector, KanR	Novagen
pKD3	Template plasmid for FRT-flanked cat cassette	[2]
pKD46	Encodes recombineering phage lambda Red genes	[2]
pCP20	Encodes the yeast Flp recombinase gene	[3]
pSTL6	pET28b encoding C-terminal His-tagged *E. coli* BioH	[1]
pSTL42	pET28b encoding *E. coli* BioH plus His_6-tagged *E. coli* BioC	Lab stock
pXC.039	pET28b encoding C-terminal His-tagged *P. aeruginosa* BioH	This study
pXC.042	pET28b encoding C-terminal His-tagged *P. aeruginosa* BioC	This study

pXC.040	A 1,000 bp PCR fragment containing the *P. aeruginosa* *bioD*::Gm^r cassette in pK19mobsacB, Gm^r, Kan^r	This study

Table S2. Oligonucleotides (5'-3')

BioH-F	BioH Forward	ATACTACCATGGATGCGTGACCACTTGATCCTGTTGCCGGGCT
BioH-R	BioH Reverse	ACTACTCGAGGGCATCGTCACCCTCGCGAAGGAATCTCA
BioC-F	BioC Forward	ATACTACCATGGATGCCTGACGATTCCTCG
BioC-R	BioC Reverse	ACTACTCGAGGGAATCCTTGCGCAGCAC
P1	P1 priming site for *E. coli* genomic bioH-His$_6$	CGAGTTTTGTCACCTGCTGGTGGCGTTGAAGCAGAGGGTGCATCACCATCACCA TCACTAATGTGTAGGCTGGAGCTGCTTC
P2	P2 priming site for *E. coli* genomic bioH-His$_6$	GCGAGTCAGTAAAGTTCTGTCTCGCCATTTCAAAAGCCACCATATGAATATCCT CCTTA
oXC48	UpbioD Forward (EcoRI)	ACATGAATTCCACAACCTCAACCCCGGGC
oXC49	DownbioD Reverse (HindIII)	ATACAAGCTTCGCGTTGAGGCCACTGACGAAA
oXC50	DownbioD Forward (BamHI)	TTCACCCTGGTGGATCCGGAGGGCGC
oXC51	UpbioD Reverse (BamHI)	GCGCCCTCCGGATCCACCAGGGTGAA
oXC53	*E. coli bioH* Reverse	GCGAGTCAGTAAAGTTCTGTCTCGCCATTTC
oXC54	*E. coli bioH* Forward	AACTCAGTGATGATTTTCAGCGTAC

oXC92	PAO1 *bioC* Forward (q-RT)	TTCGTCCACGTCAATCGCT
oXC93	PAO1 bioC Reverse (q-RT)	GCCTTGAGTTCGTGGGTCA
oXC116	*E. coli* 16S Forward (q-RT)	TACCGCATAACGTCGCAAGA
oXC117	*E. coli* 16S Reverse (q-RT)	TTCCAGTGTGGCTGGTCATC
oXC118	*E. coli bioH* Forward (q-RT)	ATTGCGTTAACCCATCCCGA
oXC119	*E. coli bioH* Reverse (q-RT)	GTTTGTAACGCCAGGAACCG
oXC124	*E. coli bioC* Forward (q-RT)	CGACGTTCGATCTTGCATGG
oXC125	*E. coli bioC* Reverse (q-RT)	GATAATGCACGCCGTTCAGC
oXC126	PAO1 bioH::His_6-Tet Forward	ATATAGAATTCAACTCGACGATAACCTGCCGCGCGATA
oXC131	PAO1 bioH::His_6-Tet Reverse	ATGCAGGAGCCGGTCCTCGT
oXC184	PAO1 *bioH* Forward (q-RT)	TGCGTGACCACTTGATCCTG
oXC185	PAO1 *bioH* Reverse (q-RT)	CGGCAGGTTATCGTCGAGTT
oXC188	PAO1 *16S* Forward (q-RT)	CAAGGCGACGATCCGTAACT
oXC189	PAO1 16S Reverse (q-RT)	ATCAGGCTTTCGCCCATTGT
oXC201	pET28b Kan Forward (q-RT)	CGGTTTGGTTGATGCGAGTG
oXC202	pET28b Kan Reverse (q-RT)	GTGACGACTGAATCCGGTGA

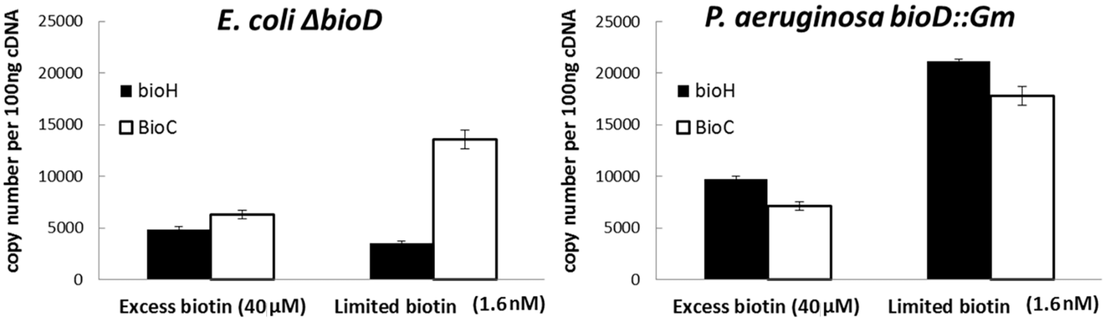

Figure S1. Transcriptional profiles of *bioH* and *bioC* in an *E. coli* Δ*bioD* (strain STL111) and in *P. aeruginosa bioD::Gm* (strain XC.059). Cultures were grown to mid-log phase in defined medium supplemented with excess biotin (40 μM) or limited biotin (1.6 nM), respectively. Absolute transcript values for each gene were obtained individually. Error bars indicate standard deviation from three independent experiments. The amplification efficiency for each pair of primers was evaluated (Materials and Methods). A linear equation of "Ct-value vs gene concentration" for *bioC* and *bioH* was generated with the corresponding primer pairs.

Supplemental References

1 Lin, S., Hanson, R. E. & Cronan, J. E. Biotin synthesis begins by hijacking the fatty acid synthetic pathway. *Nat Chem Biol* **6**, 682-688,

2 Datsenko, K. A. & Wanner, B. L. One-step inactivation of chromosomal genes in *Escherichia coli* K-12 using PCR products. *Proc Natl Acad Sci U S A* **97**, 6640-6645.

3 Cherepanov, P. P. & Wackernagel, W. Gene disruption in *Escherichia coli*: TcR and KmR cassettes with the option of Flp-catalyzed excision of the antibiotic-resistance determinant. *Gene* **158**, 9-14 (1995).
